# Expression of DENDRIN in several glomerular diseases and correlation to pathological parameters and renal failure - preliminary study

**DOI:** 10.1186/s13000-018-0767-z

**Published:** 2018-11-20

**Authors:** Maja Mizdrak, Katarina Vukojević, Natalija Filipović, Vesna Čapkun, Benjamin Benzon, Merica Glavina Durdov

**Affiliations:** 1 Department of Nephrology and Hemodialysis, University Hospital Centre Split, Šoltanska 1, 21000 Split, Croatia; 2 Department of Anatomy, Histology and Embryology, University of Split School of Medicine, Split, Croatia; 3 Department of Nuclear Medicine, University Hospital Centre Split, Split, Croatia; 4 Department of Pathology, Forensic medicine and Cytology, University Hospital Centre Split, Split, Croatia; 5 University of Split School of Medicine, Split, Croatia

**Keywords:** Dendrin, IgA glomerulonephritis, Podocythopathies, Renal function, Immunohistochemistry

## Abstract

**Background:**

In glomerular injury dendrin translocates from the slit diaphragm to the podocyte nucleus, inducing apoptosis. We analyzed dendrin expression in IgA glomerulonephritis and Henoch Schönlein purpura (IgAN/HSP) versus in podocytopathies minimal change disease (MCD) and focal segmental glomerulosclerosis (FSGS), and compared it to pathohistological findings and renal function at the time of biopsy and the last follow-up.

**Methods:**

Twenty males and 13 females with median of age 35 years (min-max: 3–76) who underwent percutaneous renal biopsy and had diagnosis of glomerular disease (GD) were included in this retrospective study. Fifteen patients had IgAN/HSP and eighteen podocytopathy. Control group consisted of ten patients who underwent nephrectomy due to renal cancer. Dendrin expression pattern (membranous, dual, nuclear or negative), number of dendrin positive nuclei and proportion of dendrin negative glomeruli were analyzed.

**Results:**

In GD and the control group significant differences in number of dendrin positive nuclei and proportion of dendrin negative glomeruli were found (*P* = 0.004 and *P* = 0.003, respectively). Number of dendrin positive nuclei was higher in podocytopathies than in IgAN/HSP, 3.90 versus 1.67 (*P* = 0.028). Proportion of dendrin negative glomeruli correlated to higher rates of interstitial fibrosis (*P* = 0.038), tubular atrophy (*P* = 0.011) and globally sclerotic glomeruli (*P* = 0.008). Dual and nuclear dendrin expression pattern were connected with lower rate of interstitial fibrosis and tubular atrophy than negative dendrin expression pattern (*P* = 0.024 and *P* = 0.017, respectively). Proportion of dendrin negative glomeruli correlated with lower creatinine clearance (CC) at the time of biopsy and the last follow-up (*P* = 0.010 and *P* < 0.001, respectively). Dendrin expression pattern correlated to CC at the last follow-up (*P* = 0.009), being lower in patients with negative than nuclear or dual dendrin expression (*P* = 0.034 and *P* = 0.004, respectively).

**Conclusion:**

In this pilot study the number of dendrin positive nuclei was higher in podocytopathies than in inflammatory GD. Negative dendrin expression pattern correlated to chronic tubulointerstitial changes and lower CC, which needs to be confirmed in a larger series.

## Background

Dendrin is a proline - rich protein of still unclear function that was originally identified in telencephalic dendrites of sleep-deprived rats [1]. Apart from the brain, dendrin is found only in the kidneys, linearly expressed in podocytes along glomerular capillary loops [2]. As an integral part of the slit diaphragm (SD) complex, dendrin contributes to the regulation of podocyte function [3]. Dendrin has a possible role in the glomerular filtration, because it directly binds to nephrin and CD2-associated protein [2–5]. These proteins interact with p85 regulatory subunit of phosphatidylinositol 3-kinase, stimulating anti-apoptotic AKT signaling [3]. In response to glomerular injury and upregulated TGF-β, dendrin relocates from the membrane to the nucleus, thereby promoting apoptosis [3, 6]. Nuclear dendrin acts as a transcriptional factor of cytosolic enzyme cathepsin L, which proteolyzes CD2-associated protein, thereby increasing the apoptotic susceptibility to pro-apoptotic TGF-β [7]. On the other side, cathepsin L cleaves the regulatory GTP-ase dynamin and actin-associated adapter synaptopodin, causing a reorganization of the podocyte microfilament system, resulting in proteinuria [5, 7].

During fetal development of human kidney, dendrin is first detected at the *capillary loop stage* and it is never expressed in podocyte nuclei [2, 3, 8]. Due to this fact, nuclear relocation of dendrin in glomerular diseases (GD) is possible marker of disease [6]. The expression of dendrin in podocyte nuclei is found in small studies of different GD: FSGS, lupus nephritis (LN), membranous nephropathy (MN) and IgAN [6, 7]. Interestingly, significant proportion of dendrin positive nuclei was not found in MCD [2]. According to Kodama et al., large number of nuclear dendrin in IgAN could be an indicator of disease activity and its progression to glomerulosclerosis [7].

The aim of our pilot study was to evaluate possible difference in dendrin expression in inflammatory and non-inflammatory GD. We analyzed 11 IgAN, 4 HSP and 18 podocytopathies (11 primary FSGS and 7 MCD) and correlated with histological parameters and renal function at the time of the biopsy and the last follow-up.

## Methods

**Fig. 1:**
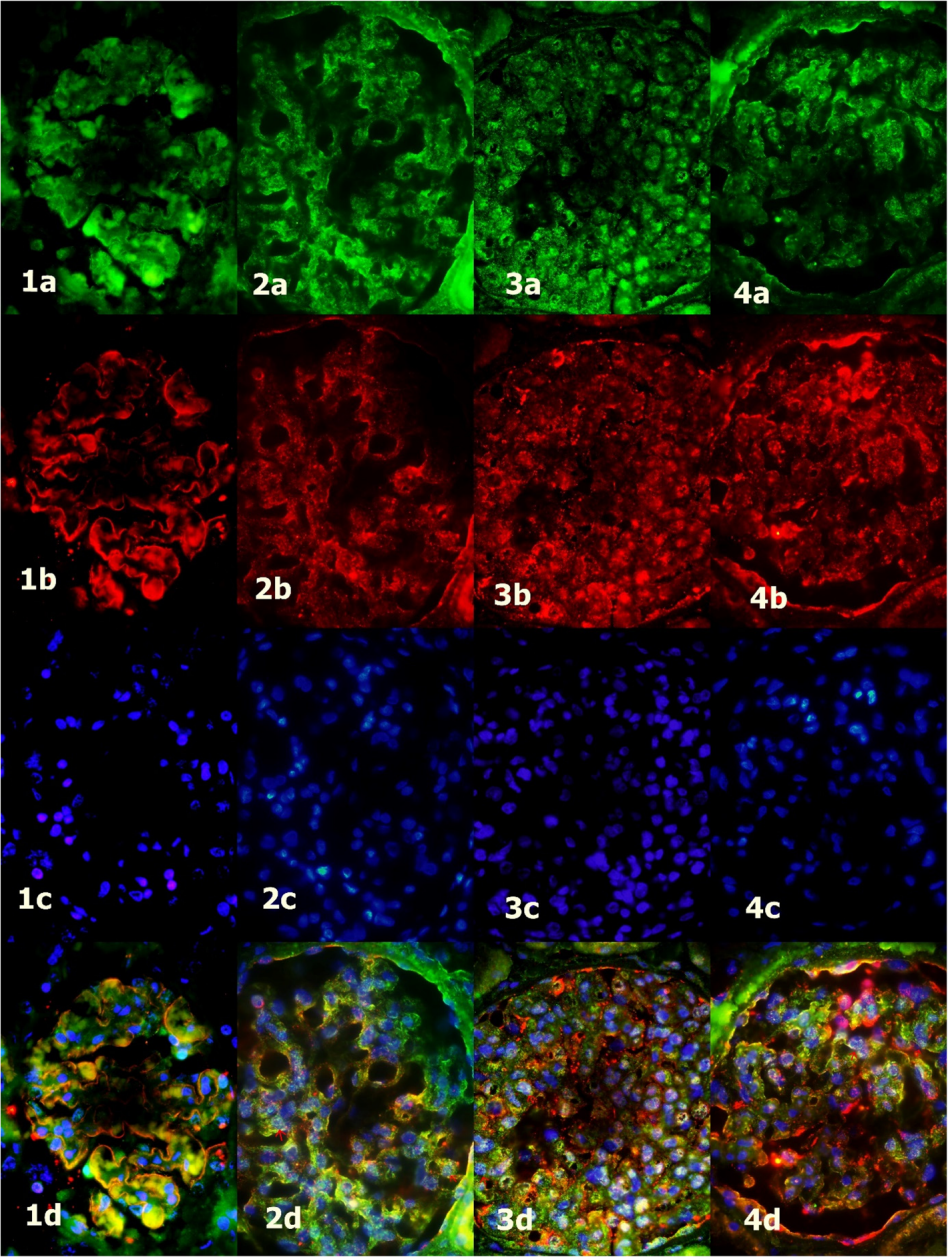
Nuclear dendrin expression (arrows) in the control group (1) and glomerular diseases (2 - MCD, 3 - IgAN/HSP, 4 - FSGS); **a** - dendrin (green), **b** - nephrin (red), **c** - DAPI (blue), **d** - merge; fluorescence microscope, immunofluorescence staining, 400 x

Thirty-three patients whose percutaneous renal biopsy was performed from 1996 to 2015 in University Hospital Centre Split, Split, Croatia were enrolled in this study. Paraffin blocks of their biopsies were collected from the Department of Pathology and clinical data at the time of biopsy and the last follow-up in 2017 were collected from the hospital records. All biopsies were analyzed by light, immunofluorescence and electron microscopy making the diagnoses of IgAN, HSP, MCD or primary FSGS. Inclusion criteria were enough material in the paraffin block for immunohistochemical analysis and complete clinical data. The patients without adequate laboratory findings or paraffin blocks were excluded. In control group we analyzed glomeruli from ten patents with normal renal function, who underwent nephrectomy due to clear cell renal carcinoma. Five micrometer thin slides were cut from the paraffin blocks. Indirect immunohistochemistry was performed in Benchmark (Ventana Medical Systems, Inc. USA). The primary antibody was rabbit polyclonal anti-dendrin (Abcam, Cambridge, United Kingdom) diluted at 1:200 and incubated for 1 h. Secondary antibody was Optiview Detection kit (Ventana Medical Systems, Inc. USA) and incubation lasted 30 min. In immunofluorescence we used rabbit anti-dendrin (Abcam, Cambridge, United Kingdom), diluted at 1:200 and goat anti-nephrin antibody (G-20 Santa Cruse Biotechnology, Inc. Heidelberg, Germany), diluted at 1:300. They were applied simultaneously and left overnight in a humidified chamber at room temperature. The secondary antibodies were donkey anti-goat IgG (Alexa fluor 594 Molecular Probes Life Technologies, Oregon, USA), diluted at 1:300 and donkey polyclonal anti-rabbit IgG (Alexa fluor 488 Molecular Probes Life Technologies, Oregon, USA), diluted at 1:300. After washing in phosphate-buffered saline (PBS), DAPI (4′,6-diamidino-2-phenylindole) was applied and washed again. Stained sections were viewed and photographed, using a BX51 microscope (Olympus, Tokyo, Japan) equipped with a DP71 digital camera (Olympus), and processed with CellA Imaging Software for Life Sciences Microscopy (Olympus). Dendrin translocation to nuclei was presented by co-localization of dendrin, nephrin and DAPI in the same cell on immunofluorescence (Fig. 1).

**Fig. 2:**
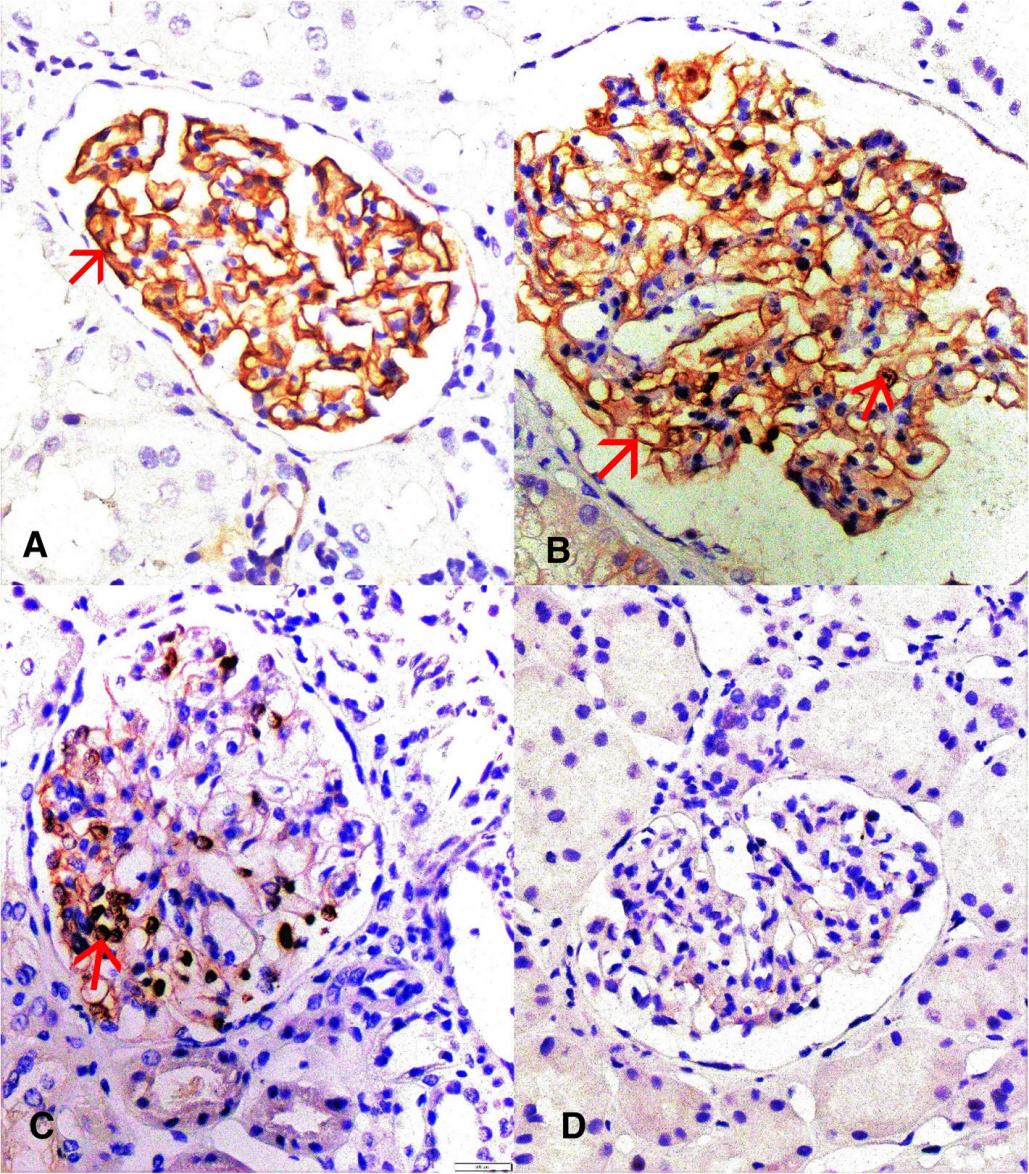
Dendrin expression pattern may be membranous (**a**), dual (**b**), nuclear (**c**) or negative (**d**); light microscope, indirect immunohistochemistry, 400 x

Nuclear expression of dendrin was counted on immunohistochemical slide on the light microscope Olympus BX51, Olympus, Tokyo, Japan. Median was 6 glomeruli (min-max: 1–34) per slide. The same was done on immunofluorescence microscope. Since there was no statistical difference in number of dendrin positive nuclei between these two methods, only the results of the immunohistochemistry were presented in the further text. Number of dendrin positive nuclei was divided with the number of analyzed glomeruli and thus median (min-max) was calculated for each patient. Number of dendrin negative glomeruli was divided with number of analyzed glomeruli to get their proportion. Control slides were analyzed on one low magnification field (7 glomeruli in average) and analyzed on the same way. A pattern of dendrin expression in each glomerulus was assigned as membranous, dual, nuclear or negative. Only membranous staining was assigned as membranous, simultaneously positive membranous and nuclear staining as dual, only nuclear staining as nuclear and both negative membranous and nuclear staining with positive external control as negative (Fig. 2).

Statistical analysis was performed using Statistical Package for the Social Sciences (SPSS) software (version 19 for Windows; SPSS Inc., Chicago, Illinois, USA). Statistical significance was set at *P* < 0.05, and all confidence intervals (CI) were at the 95% level. Spearman’s correlation coefficient (Rho) was used to calculate degree of connection. Statistical significance of the difference in categorical characteristics of several independent variables was calculated by using χ^2^ test. Fisher’s exact test was used in the analysis of contingency tables when tested sample sizes were small. Analysis of statistical significance of differences in several numerical variables was performed with the Kruskal-Wallis test and of differences between two groups with the Mann-Whitney test. The study was performed according to the Helsinki Declaration. Informed signed consent form was obtained from each patient (or parents if a child).

## Results

In the study there were 20 (61%) males, with median of age 38.5 (min-max: 3–76 years) and 13 (39%) females, with median of age 35 (min-max: 4–62 years). Eleven (33.33%) patients had IgAN, four (12.12%) HSP, seven (21.21%) MCD and eleven (33.33%) FSGS. The distribution according to the type of glomerular disease and clinical parameters is presented in Table 1. Due to small numbers, IgAN and HSP, and MCD and FSGS were analyzed together.

**Table 1:** Distribution of 33 patients according to the type of glomerular disease and clinical parameters

Clinical parameter		Patients with glomerular disease				
		Inflammatory		Non-inflammatory		*P*
		IgAN (*n* = 11)	HSP (*n* = 4)	MCD (*n* = 7)	FSGS (*n* = 11)	
Hematuria	yes	4	4	1	4	0.169^a^
Hypertension	yes	7	1	5	9	0.163^a^
Proteinuria	≥ 3400 (mg/dU/24 h)	6	2	6	9	0.126^a^
Creatinine clearance (ml/min/1.73m^2^) median value (min-max)	At the time of biopsy	96 (27–150)	84 (25–170)	89 (27–212)	62 (30–162)	0.800^b^
	At the last follow-up	59 (6–147)	75.5 (47–148)	103 (63–180)	51 (7–92)	0.942^b^

^a^Fisher’s exact test

^b^Mann Whitney test

Patients with several GD were not significantly different in any clinical parameters. In further analysis types of GD and the control group were analyzed according to median of number of nuclear dendrin expression and proportion of dendrin negative glomeruli (Table 2).

**Table 2:** Number of dendrin positive nuclei and proportion of dendrin negative glomeruli in glomerular diseases

Dendrin expression	Glomerular disease (*N* = 33)				
	Inflammatory (*n* = 15)	Non-inflammatory (*n* = 18)			
	IgAN + HSP	MCD	FSGS	Control	*P* ^a^
Dendrin-positive nuclei^b^	1.67 (0.00–5.60)	5.90 (2.00–23.10)	2.80 (0.00–9.50)	0.50 (0.00–2.20)	0.004
Proportion of dendrin negative glomeruli^b^	0.50 (0.00–1.00)	0.00 (0.00–0.06)	0.27 (0.00–1.00)	0.00 (0.00–0.00)	0.003

^a^Kruskal Wallis test

^b^median (min-max)

In IgAN/HSP median number of dendrin positive nuclei was 1.67 (min - max: 0.00–5.60) and in podocytopathies 3.90 (min – max: 0.00–23.10), (*P* = 0.028, Z = 2.2, *r* = 0.38, 95% CI 0.12–4.34). In further analysis MCD and FSGS were analyzed separately. Nuclear dendrin expression was different in IgAN/HSP, MCD, FSGS and control group (χ^2^ = 13.10; *P* = 0.004). Median nuclear dendrin expression was higher in MCD than IgAN/HSP (*P* = 0.033) and in MCD than the control group (*P* = 0.003). No difference was found between IgAN/HSP and FSGS (*P* = 1.000). Median of nuclear dendrin expression was not significantly different in MCD and FSGS (*P* = 0.363). Proportion of dendrin negative glomeruli was statistically different between GD (χ^2^ = 13.60; *P* = 0.003). In IgAN/HSP and FSGS proportions of dendrin negative glomeruli were higher than in the control group (*P* = 0.015 and *P* = 0.035, respectively). There was no difference between IgAN/HSP and FSGS (*P* = 1.000). Difference was not found between MCD and control group (*P* = 1.000).

CC at the time of biopsy did not significantly correlate to median of dendrin positive nuclei (Rho = 0.150, *P* = 0.403). According to Spearman’s coefficient of correlation, CC at the time of biopsy and proportion of dendrin negative glomeruli showed negative correlation (Rho = − 0.444; *P* = 0.010). CC at the last follow-up significantly correlated to proportion of dendrin negative glomeruli (Rho = − 0.626; *P* < 0.001) and to median of dendrin positive nuclei (Rho = 0.433, *P* = 0.012). No difference was found in correlation between the level of proteinuria and median of dendrin positive nuclei (Rho = 0.324, *P* = 0.066), as well as proteinuria and proportion of dendrin negative glomeruli (Rho = − 0.284, *P* = 0.109).

Number of dendrin positive nuclei in IgAN/HSP was correlated to parameters of Oxford classification, but significant difference was not found (Table 3).

**Table 3:** Number of dendrin positive nuclei according to Oxford classification of IgAN

Parameter	Number of cases	Dendrin positive nuclei median (min-max)	*P* ^*^
Mesangial hypercellularity (M)	M0 (*n* = 2)	1.50 (0.00–4.60)	0.941
	M1 (*n* = 13)	2.00 (0.00–5.60)	
Endocapillary proliferation (E)	E0 (*n* = 11)	1.50 (0.00–5.60)	0.549
	E1 (*n* = 4)	2.40 (0.00–3.85)	
Segmental sclerosis (S)	S0 (*n* = 7)	2.30 (0.00–5.60)	0.376
	S1 (*n* = 8)	1.50 (0.00–4.60)	
Tubular atrophy/interstitial fibrosis (T)	T0 (*n* = 15)	1.70 (0.00–5.60)	/
	T1 (*n* = 0)	/	
	T2 (*n* = 0)	/	

^*^Mann-Whitney test

In eight IgAN samples with crescents median of dentrin positive nuclei was 1.58 (min – max: 0.00–3.85) and in seven IgAN samples without crescents 2.30 (min – max: 0.00–5.60) (*P* = 0.479). Proportion of dendrin negative glomeruli in the first group was 0.50 (min-max: 0.00–1.00), and in the former 0.33 (0.00–1.00) (*P* = 0.668). According to International Study of Kidney Disease in Children (ISKDC) classification, three patients with HSP were grade 3, and one grade 6.

Dendrin expression pattern was assigned to each patient according to predominate finding in whole slide, and it was nuclear in nine (27.27%), dual in sixteen (48.48%) and negative in eight (24.24%) patiens. There was no difference between IgAN/HSP and podocytopathies in distribution of dual or nuclear dendrin expression pattern (*P* = 0.299). Also there was no difference in dual/nuclear versus negative expression pattern (*P* = 0.418). Dendrin expression pattern in 33 patients was correlated to pathohistological findings and clinical parameters of renal function (Table 4).

**Table 4:** Dendrin expression pattern in relation to pathohistological and clinical parameters

Parameter	Pattern of dendrin expression in 33 patients with glomerular diseases			*P* ^a^
	Nuclear (*N* = 9)	Dual (*N* = 16)	Negative (*N* = 8)	
Interstitial fibrosis	0.05 (0.00–0.35)	0.01 (0.00–0.20)	0.10 (0.00–0.20)	0.024
Tubular atrophy	0.01 (0.00–0.35)	0.01 (0.00–0.20)	0.10 (0.01–0.25)	0.017
Globally sclerotic glomeruli	0.04 (0.00–0.35)	0.00 (0.00–0.21)	0.17 (0.00–0.50)	0.056
Creatinine clearance at the time of biopsy (ml/min/1.73m^2^)^b^	53 (27–118)	108 (25–212)	58 (27–150)	0.073
Creatinine clearance at the last follow-up (ml/min/1.73m^2^)^b^	68 (10–109)	91 (17–180)	11.5 (6–95)	0.009
Proteinuria (mg/dU/24 h)^b^	6380 (1460–18,677)	5375 (100–28,254)	4150 (63–5250)	0.208

aKruskal-Wallis test

^b^Median (min-max)

Dendrin expression pattern significantly correlated to median of CC at the last follow-up (χ^2^ = 9.30, ƞ^2^ = 0.29; *P* = 0.009). Mann-Whitney test in post-hoc analysis did not find any difference between the patients with nuclear or dual expression (Z = 1.20; *P* = 0.234). In patients with negative dendrin expression pattern, median of CC was significantly lower than in patients with nuclear expression pattern (Z = 2.10; *r* = 0.52; *P* = 0.034) and for 79.5 lower than in patients with dual expression pattern (Z = 2.80, *r* = 0.59, *P* = 0.004). CC at the time of biopsy and proteinuria were not significantly different between dendrin expression patterns (χ^2^ = 5.20; *P* = 0.073 and χ^2^ = 3.1, *P* = 0.208, respectively).

According to Spearman’s coefficient of correlation, proportion of globally sclerotic glomeruli and number of dendrin positive nuclei showed negative correlation (Rho = − 0.409, *P* = 0.018). Significant correlations between number of dendrin positive nuclei and interstitial fibrosis (Rho = − 0.167, *P* = 0.353) or tubular atrophy (Rho = − 0.277, *P* = 0.119) were not found. Statistically significant positive Spearman’s correlations were found in proportions of interstitial fibrosis (Rho = 0.363, *P* = 0.038), tubular atrophy (Rho = 0.437, *P* = 0.011) and globally sclerotic glomeruli (Rho = 0.451, *P* = 0.008) with proportion of dendrin negative glomeruli.

**Fig. 3:**
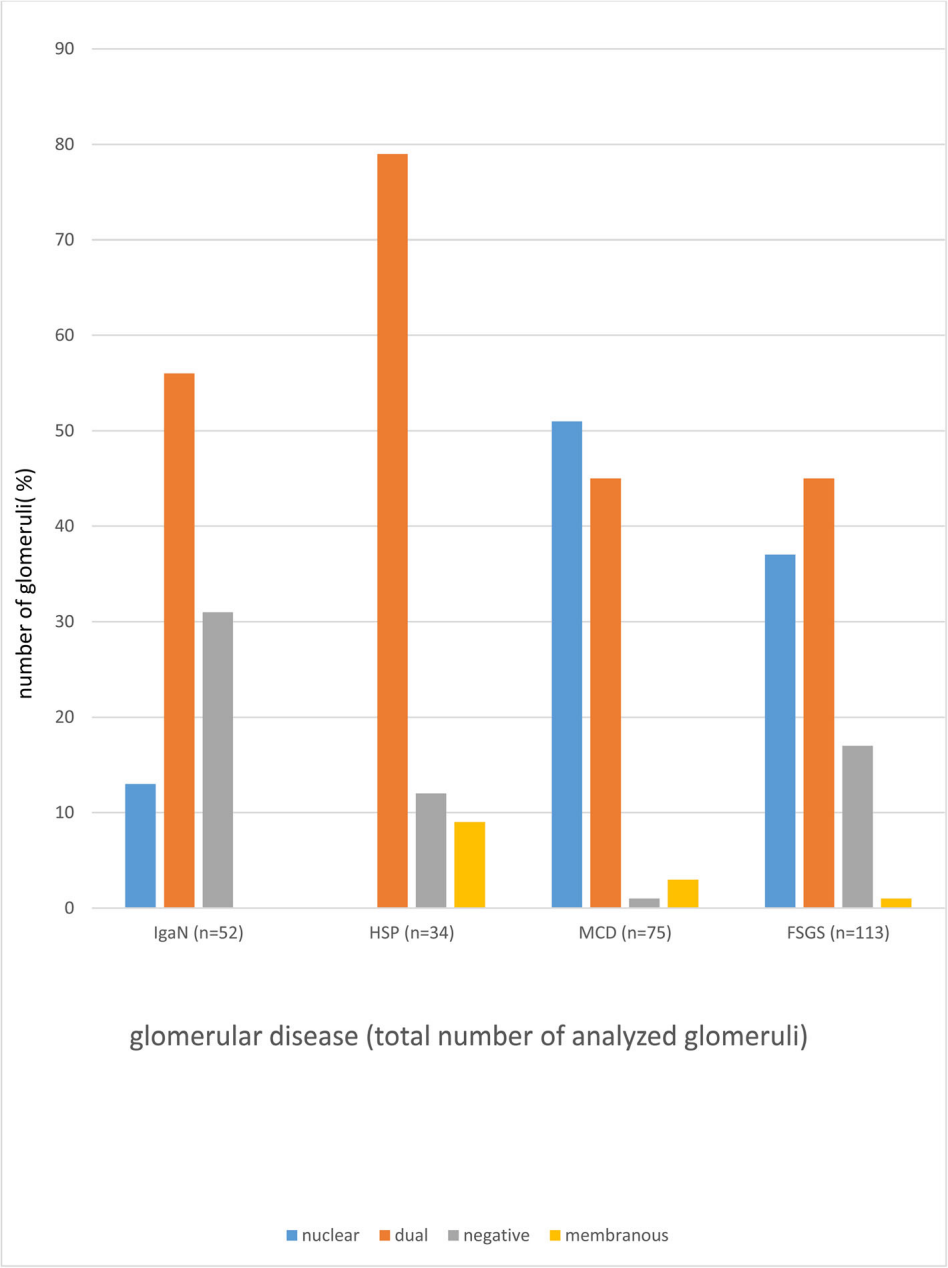
Distribution of dendrin expression pattern in total number of glomeruli in analyzed types of glomerular diseases

At last, dendrin expression pattern in all glomeruli was analyzed and different distribution according to type of GD was found (χ^2^ = 52; *P* < 0.001) (Table 5, Fig. 3).

**Table 5:** Distribution of dendrin expression pattern in total number of glomeruli in analyzed glomerular diseases

	Total number of analyzed glomeruli in each type of glomerular diseases *n* (%)				
Dendrin expression pattern	IgAN *n* = 52	HSP *n* = 34	MCD *n* = 75	FSGS *n* = 113	*P* ^*^
Nuclear	7 (13)	0 (0)	38 (51)	42 (37)	< 0.001
Dual	29 (56)	27 (79)	34 (45)	51 (45)	
Negative	16 (31)	4 (12)	1 (1)	19 (17)	
Membranous	0 (0)	3 (9)	2 (3)	1 (1)	

*χ^2^ test

In IgAN nuclear pattern was found in 13% glomeruli and none in HSP (χ^2^ = 9.60; *P* < 0.001). In MCD there was 3.9 times more glomeruli with nuclear dendrin expression pattern than in IgAN, but only one glomerulus with negative dendrin expression (χ^2^ = 28; *P* < 0.001). IgAN had 3.1 times less glomeruli with nuclear dendrin expression than FSGS (χ^2^ = 19.60; *P* < 0.001). Nuclear dendrin expression was found in MCD, in contrast to HSP (χ^2^ = 28.20; *P* < 0.001). Dual pattern was 1.8 times more common in HSP than FSGS (χ^2^ = 19.60; *P* < 0.001). In MCD one glomerulus had negative expression pattern and in FSGS 17% glomeruli (χ^2^ = 12.10; *P* < 0.001).

## Discussion

During glomerular injury, dendrin nuclear translocation promotes podocyte apoptosis, which is the main pathological mechanism of chronic kidney failure [9]. This pathway is strictly controlled by regulatory pro- and antiapoptotic molecules [10]. In an experimental model, the translocation from membrane to nucleus might be temporarily presented as a dual subcellular dendrin distribution [3]. In our immunohistochemical pilot study of human glomerular diseases, we have noticed not only dual, but also negative dendrin expression in several glomeruli. Dendrin expression pattern was different in analyzed types of glomerular diseases and higher proportion of dendrin negative glomeruli significantly correlated to lower creatinine clearance. We accentuate a term of *dendrin negative glomerulus* and suggest that dendrin expression might be irreversibly switched off in chronically damaged glomeruli. We assume that higher proportion of such glomeruli is possibly associated with podocyte loss and thereby is prognostically relevant to the kidney function. In our study, higher proportion of dendrin negative glomeruli has been significantly correlated to lower CC both at the time of biopsy and the follow-up, and for our best knowledge it is the first such data in the literature. Becherucci et al. explained the pathway of glomerulosclerosis in which dendrin has an important role. The limited podocyte death induced hypertrophy of survived podocytes; if podocyte loss had increased over a certain threshold, further step was the activation of parietal epithelial cells and their differentiation to podocytes, and if this compensatory mechanism was not efficient, induction of sclerosis followed [11]. In our study there were positive statistically significant Spearman’s correlations between proportion of dendrin negative glomeruli and rate of interstitial fibrosis, tubular atrophy and globally sclerotic glomeruli. Higher proportion of globally sclerotic glomeruli was also correlated to lower number of dendrin positive nuclei what is in accordance with the literature. Kodama et al. found in the IgAN study that the number of dendrin positive nuclei was significantly higher in the renal biopsy specimens with only a few sclerotic glomeruli [7].

According to previously reported small studies, nuclear dendrin relocation occurred in IgAN, FSGS, MN and LN [6, 7]. In the study of three cases of FSGS and MN and four cases of LN, Asunama et al. found significantly higher number of dendrin positive nuclei than in control (five cases) and MCD (three cases), in which a few dendrin-positive nuclei still were present [6]. Dunér et al. analyzed five patients with MCD and found cytoplasmic dendrin expression in areas with foot process effacement, but not in podocyte nuclei [2]. We used light and immunofluorescence microscopy and confirmed that number of dendrin positive nuclei was not significantly different between two analyses. In our study clear nuclear relocation of dendrin in MCD was found. MCD is also podocytopathy caused by immunological factors despite regular light or immunofluorescence microscopy presentation [12]. Therefore, we assume that dendrin relocation is the response to glomerular injury, unrelated to type of the glomerular disease. In the future, a larger study would be necessary to examine dendrin nuclear expression in cohort of MCD patients of the same age.

Recent insights have defined the central role of the podocyte as both the regulator of glomerular development as well as the determinant of progression to glomerulosclerosis in podocytopathies [13]. In inflammatory GD - IgA nephropathy/HSP mesangial cell activation is an initial consequence of IgA deposition, but mesangial-podocyte crosstalk leads to indirect podocyte injury which has cytoskeletal and signaling consequences, ie. indirect podocyte injury also contributes to glomerular damage observed in IgAN [14].

We have examined dendrin expression pattern in all glomeruli of each type of glomerular diseases and have found the significant difference. In HSP, the disease with usually an acute clinical presentation, predominantly dual pattern of dendrin expression has been found, without nuclear expression and more membranous expression than in other GD. In IgAN dual and negative patterns were found and in podocytopathies predominantly nuclear and dual expression. Only one dendrin negative glomerulus was detected in MCD, in contrast to IgAN and FSGS (31 and 17%, respectively). These changes could be explained by the differences in the pathophysiological mechanisms, the clinical course and dynamics of a particular disease.

## Conclusion

Relocation of dendrin from membrane to nucleus is a dynamic, gradual and multi-level process controlled and affected by numerous factors and signal pathways. We have found that dendrin translocation occurs in different types of glomerular diseases, including MCD, and dendrin expression presents in different patterns. The proportion of dendrin negative glomeruli could be a novel adverse prognostic factor of the kidney function. We propose that dendrin translocation and its correlation with kidney failure and pathological parameters should be interpreted in the context of dendrin expression pattern, not only as an absolute number of dendrin positive nuclei.

## Abbreviations

CC: creatinine clearance
DAPI: 4′,6-diamidino-2-phenylindole
FSGS: focal segmental glomerulosclerosis
GD: glomerular disease
GTP: hydrolyze guanosine triphosphate
HSP: Henoch-Schӧnlein purpura
IgAN: IgA nephropathy
LN: lupus nephritis
max: maximal
MCD: minimal change disease
min: minimal
MN: membranous nephropathy
PBS: phosphate-buffered saline
SD: slit diaphragm
TGB-β: transforming growth factor beta
